# Exploring the relationship between morphea and malignancy: a decade-long single-center study of 204 patients

**DOI:** 10.1007/s00403-024-03357-7

**Published:** 2024-09-11

**Authors:** Keren Lyakhovitsky, Giovanni Damiani, Daniel Mimouni, Anna Aronovich

**Affiliations:** 1 Division of Dermatology, Rabin Medical Center, Petach Tikva, Israel; 2 School of Medicine, Faculty of Medical and Health Sciences, Tel Aviv University, Tel Aviv, Israel; 3 UOC Maxillofacial Surgery and Dentistry, Ospedale Maggiore Policlinico, Fondazione IRCCS Ca’ Granda, Milan, Italy; 4 Department of Biomedical, Surgical and Dental Sciences, University of Milan, Milan, Italy

**Keywords:** Localized scleroderma, Morphea, Mortality, Malignancy, Neoplasms

## Abstract

The association between systemic scleroderma and malignancy is well-documented, but there is limited data on the relationship between morphea and malignancy. This study aims to assess the incidence and types of malignancies in morphea patients, comparing demographics, clinical characteristics, treatments, and outcomes between those with and without malignancy. We conducted a retrospective study of 204 morphea patients treated at Rabin Medical Center between 2012 and 2023. Data on demographics, clinical subtypes, comorbidities, treatments, and outcomes were collected. Patients were categorized based on malignancy status and the timing of malignancy relative to their morphea diagnosis. Among the 204 patients (154 women and 50 men, mean age 53.7 ± 20 years), 47 (23%) developed malignancies. In 29 patients (61.7%), malignancy occurred before the onset of morphea; in 23 patients (48.9%), it occurred after morphea. Five patients (10.6%) had malignancies both before and after the diagnosis of morphea. Patients with malignancy were significantly older than those without (64.7 ± 15.1 years vs. 50.3 ± 20 years, *p* < 0.0001). The all-cause mortality rate was higher in the malignancy group compared to those without malignancy (23.4% vs. 3.8%, *p* = 0.00002). Moreover, mortality was higher in patients whose malignancy occurred after morphea than in those whose malignancy preceded morphea (26% vs. 17.2%). The most common post-morphea malignancies in our cohort included non-melanoma skin cancer, cervical cancer, breast cancer, stomach cancer, and lung cancer. The most common pre-morphea malignancies included breast cancer, non-melanoma skin cancer, colon cancer, prostate cancer, and testicular cancer. This study suggests potential associations between morphea and malignancies, influenced by patient age, sequence of diagnosis, and treatment regimens. Further control studies are needed to explore these relationships more definitively.

## Introduction

Morphea (localized scleroderma), a rare, chronic inflammatory connective tissue disorder that manifests with inflammation and fibrosis affecting the skin and underlying soft tissue, with possible extension to adjacent structures (e.g., lungs, gastrointestinal tract) [1]. Despite being primary recognized as a condition confined to the skin, some subtypes of morphea exhibit extra-cutaneous manifestations, involving the musculoskeletal and central nervous systems [1]. The annual incidence of morphea varies from 4 to 27 new cases per million, with approximately two-thirds occurring in adults [1]. There are two incidence peaks: between ages 2 to 14 and in the 5th decade of life [1]. Morphea predominantly affects females, with a female-to-male ratio of 4:1, and is most common among Caucasians, followed by Hispanics and Latin Americans [2].

The five main clinical types of morphea include limited, generalized, linear, deep, and mixed, with plaque-type and generalized morphea being prevalent in adults, while the linear form is more common in children [3].

Morphea’s pathogenesis involves a complex interplay of genetic predisposition, vascular dysfunction, environmental triggers, and autoimmune dysregulation [1]. Possible triggers for morphea include cancer treatments like radiotherapy, chemotherapy, and immunotherapy [1]. It unfolds in three phases: an initial inflammatory phase, a fibrotic phase marked by excessive collagen deposition, and an atrophic phase. Th1/Th2 imbalance and the upregulation of profibrotic pathways are key factors, with an initial proinflammatory Th1/Th17-associated cytokine response followed by a shift towards Th2 cytokines during disease progression [4].

Diagnosing morphea relies primarily on clinical features, with skin biopsy reserved for atypical cases. Treatment options vary based on disease severity [5]. Topical corticosteroids are used for limited disease, while systemic corticosteroids and methotrexate are most commonly utilized in more extensive cases. Additional options for topical therapies include calcineurin inhibitors, vitamin D derivatives, and imiquimod [6]. For more severe cases, systemic treatment options encompass mycophenolate mophetil, hydroxychloroquine, retinoids, anti-interleukin-6 receptor antibody and janus kinase inhibitors [6]. Other treatment modalities include various phototherapy types and laser treatments [7]. Emerging antifibrotic and anti-inflammatory agents continue to shape the therapeutic landscape [6].

The association between morphea and malignancy is an evolving research area. While systemic sclerosis (SSc) patients are known to have an elevated susceptibility to malignancy, attributed to shared risk factors, chronic inflammation, premature immunosenescence, impaired DNA repair, and therapy-related immunosuppression, limited data is available for morphea [8]. Some studies suggest an increased risk of secondary malignancies, particularly in those with generalized disease [9]. Additionally, there have been reported cases of morphea induced by cancer treatments such as radiotherapy, chemotherapy, and immunotherapy [9]. Nevertheless, the majority of previous studies have concentrated on specific patient subgroups and types of malignancy [10, 11].

This study aimed to assess the incidence and types of malignancies among patients diagnosed with morphea. Additionally, the study sought to analyze the demographics, medical histories, clinical characteristics, treatment modalities, and mortality rates in morphea patients, distinguishing between those with and without malignancy.

## Materials and methods

### Study design

This retrospective cohort study analyzed the medical records of patients diagnosed with morphea. Patients were diagnosed, treated, and monitored in the dermatology department and outpatient clinic at the Rabin Medical Center, a tertiary care facility, from January 2012 to December 2023. The study was conducted with approval from the institutional review board (Approval Number [2021 − 1401]). Patient data were retrieved from the Hospital Chameleon System electronic medical records and subsequently reviewed.

### Study population

Inclusion criteria encompassed patients of all ages with a confirmed diagnosis of morphea, established through typical clinical presentation and histopathological confirmation with atypical presentation. Patients were required to have a minimum follow-up duration of 6 months post-diagnosis. Exclusion criteria involved patients with misdiagnoses or incomplete medical records.

### Data collection

Demographic data, comorbidities (including general conditions and malignancies), clinical parameters (comprising clinical subtypes and symptoms), treatment modalities, and follow-up durations were collected. For patients diagnosed with malignancies, details regarding the type of malignancy, timing of diagnosis relative to morphea diagnosis, and mortality rates were recorded.

### Data analysis

All patients were divided into two principal groups: individuals lacking malignancies and those with malignancies (either diagnosed before or after the onset of morphea), followed by a thorough comparative analysis. Following this, subgroups comprising patients with malignancies were further delineated into those who developed malignancy before the diagnosis of morphea and those who did so after. The time interval between diagnoses was carefully assessed.

### Statistical analysis

Categorical variables were reported as numbers and percentages, and continuous variables were presented as mean and standard deviation (SD). Chi-square tests were utilized for comparing dichotomous variables, whereas unpaired Student’s t-tests were applied for dichotomous and quantitative variables such as age and follow-up duration. All statistical tests were two-sided and conducted at a significance level of 0.05. Data analysis was performed using IBM SPSS Statistics version 25 (IBM Corp., Armonk, NY, USA).

## Results

### Patient demographics and comorbidities

Data are presented in Table 1. The study encompassed a total of 204 patients in its final analysis, after excluding 238 individuals due to misdiagnosis or uncertain diagnostic criteria, 10 due to lack of access to data, and 20 due to insufficient follow-up.

**Table 1:** Comparison of demographic characteristics and medical backgrounds in morphea patients with and without malignancy

Variable	Morphea with malignancy (*n* = 47)	Morphea without malignancy (*n* = 157)	Total (*n* = 204)	*p*-value
Female gender, n (%)	37 (78.7%)	117 (74.5%)	154 (75.5%)	0.557
Male gender, n (%)	10 (21.3%)	40 (25.5%)	50 (24.5%)	
Age at onset of morphea, years, mean (SD)	64.7±15.1	50.3±20	53.7±20	**< 0.001**
Age range	27–89	4–86	4–89	-
Time to morphea diagnosis, years, mean (SD)	2.93±5.7	3.39±5.3	3.25±5.4	0.609
**Ethnicity**,** n (%)**				
Jews	45 (95.7%)	151 (96.2%)	196 (96%)	0.913
Arabs	2 (4.3%)	6 (3.8%)	8 (4%)	
**Smoking**,** n (%)**	9 (19.1%)	27 (17.2%)	36 (17.6%)	0.758
**Comorbidities**,** n (%)**				
Obesity	13 (27.6%)	38 (24.2%)	51 (25%)	0.631
Hypertension	26 (55.3%)	52 (33.1%)	78 (38.2%)	**0.006**
Hyperlipidemia	24 (51%)	74 (47.1%)	98 (48%)	0.636
Diabetes mellitus type 2	14 (29.8%)	32 (20.4%)	46 (22.5%)	0.176
Cardiovascular	12 (25.5%)	24 (15.3%)	36 (17.6%)	0.106
Atopy	9 (19.1%)	35 (22.3%)	44 (21.5%)	0.646
**Systemic autoimmune disorders**				
Lupus Erythematosus Systemicus	0 (%)	1 (0.64%)	1 (0.49%)	1
Rheumatoid arthritis	0 (0%)	3 (1.91%)	3 (1.47%)	1
Sarcoidosis	0 (0%)	1 (0.64%)	1 (0.49%)	1
**Organ specific autoimmune disorders**				
**Endocrine**				
Diabetes mellitus type 1	0 (0%)	1 (0.64%)	1 (0.49%)	1
Dysthyroidisms	10 (21.3%)	30 (19.1%)	40 (19.6%)	0.743
Neurological (Multiple Sclerosis)	0 (0%)	2 (1.3%)	2 (1%)	1
Gastrointestinal	2 (4.3%)	4 (2.5%)	6 (2.9%)	0.543
Hematological*	0 (0%)	6 (3.8%)	6 (2.9%)	0.339
**Dermatological**				
Psoriasis	4 (8.5%)	5 (3.2%)	9 (4.4%)	0.119
Vitiligo	1 (2.1%)	4 (2.5%)	5 (2.4%)	0.870
Alopecia areata	0 (0%)	3 (1.91%)	3 (1.47%)	1
Family history of atopy	6 (12.7%)	21 (13.4%)	27 (13.2%)	0.914
Family history of rheumatological disorders	0 (0%)	7 (4.4%)	7 (3.4%)	0.356
Family history of other autoimmune disorders	2 (4.3%)	12 (7.6%)	14 (6.8%)	0.420

SD-standard deviation. *Hematological: neutropenia, IgA deficiency, Immune thrombocytopenic purpura. Statistically significant *p*-values are highlighted in bold

Among all patients, 47 developed malignancies, while 157 did not. The average time period from the onset of skin changes to the confirmed morphea diagnosis was 3.25 ± 5.4 years, indicating a significant delay without notable differences between patients with and without malignancy. Among the 204 included patients, 154 were women and 50 were men, yielding a female-to-male ratio of 3.08. The patients’ ages varied considerably, with an overall mean age at the time of morphea diagnosis of 53.7 ± 20 years. The mean age was 54.6 ± 18.6 years for women and 51.5 ± 22.4 years for men, with no significant gender differences (*p* = 0.39). Patients with malignancy were significantly older than those without (64.7 ± 15.1 vs. 50.3 ± 20, *p* < 0.0001). No differences in gender or ethnicity were observed between the groups. Except for a higher prevalence of hypertension in patients with malignancy, medical backgrounds were similar.

### Clinical characteristics, treatments, and outcomes

The data are summarized in Table 2. The clinical subtypes of morphea were distributed as follows: limited in 113 (55.4%) patients, generalized in 72 (35.3%), linear in 16 (7.8%), and deep in 3 (1.47%). Among limited morphea subtypes, atrophoderma of Pasini and Pierini was more common in patients with malignancy than in those without (8.5% vs. 1.9%, *p* = 0.029). No differences were observed in the frequency of other clinical subtypes between groups. Most patients were asymptomatic (68.2%), 25% reported itchiness (only 2 patients, or 1%, had an atopic background), and 6.8% experienced pain, with no significant differences in symptoms between groups. Topical treatments alone were administered to 45.6% of patients, with topical corticosteroids being the most frequently prescribed, given to 77.9% of patients. The most commonly employed systemic treatments included methotrexate, administered to 12.7% of patients, followed by intravenous corticosteroids (10.7%), and hydroxychloroquine (5.4%). In the comparison of systemic treatments, a lower proportion of patients in the morphea group associated with malignancy received methotrexate.

**Table 2:** Comparison of clinical characteristics, treatments, and outcomes in morphea patients with and without malignancy

Variable	Morphea with malignancy (*n* = 47)	Morphea without malignancy (*n* = 157)	Total (*n* = 204)	*p*-value
**Clinical subtype**, **n (%)**				
**Limited**				
Plaque-type morphea	23 (49%)	77 (49%)	100 (49%)	0.989
Atrophoderma of Pasini and Pierini	4 (8.5%)	3 (1.91%)	7 (3.4%)	**0.029**
Bullous	0 (0%)	3 (1.91%)	3 (1.47%)	1
Keloid	0 (0%)	3 (1.91%)	3 (1.47%)	1
**Linear**				
Morphea of the extremities	0 (0%)	4 (2.5%)	4 (2%)	0.575
Morphea en coup de sabre	0 (0%)	11 (7%)	11 (5.4%)	0.072
Progressive facial hemiatrophy (Parry-Romberg syndrome)	0 (0%)	1 (0.64%)	1 (0.49%)	1
**Generalized**	20 (42.5%)	52 (33.2%)	72 (35.3%)	0.235
**Deep**	0 (0%)	3 (1.91%)	3 (1.47%)	1
**Symptoms**				
Pain	2 (4.3%)	12 (7.6%)	14 (6.8%)	0.420
Itch	10 (21.3%)	41 (26.1%)	51 (25%)	0.502
Asymptomatic	35 (74.4%)	104 (66.3%)	139 (68.2%)	0.288
**Treatment**,** n(%)**				
**Topical**				
Corticosteroids	39 (83%)	120 (76.4%)	159 (77.9%)	0.342
Calcineurin inhibitors	1 (2.1%)	8 (5%)	9 (4.4%)	0.385
Other*	12 (25.5%)	32 (20.4%)	44 (21.5%)	0.451
Topical treatment only	18 (38.3%)	75 (47.8%)	93 (45.6%)	0.253
**Systemic**				
Oral corticosteroids	0 (0%)	3 (1.91%)	3 (1.47%)	1
IV corticosteroids	2 (4.3%)	20 (12.7%)	22 (10.7%)	0.100
Tetracyclines	2 (4.3%)	2 (1.3%)	4 (2%)	0.196
Hydroxychloroquine	3 (6.4%)	8 (5%)	11 (5.4%)	0.732
Methotrexate	2 (4.3%)	24 (15.3%)	26 (12.7%)	**0.047**
Mycophenolate Mophetil	2 (4.3%)	7 (4.4%)	9 (4.4%)	0.953
Retinoids**	1 (2.1%)	5 (3.2%)	6 (2.9%)	0.707
Other***	0 (0%)	2 (1.3%)	2 (1%)	1
**Phototherapy**				
PUVA	12 (25.5%)	27 (17.2%)	39 (19.1%)	0.202
NBUVB	7 (14.9%)	10 (6.4%)	17 (8.3%)	0.064
BBUVB	1 (2.1%)	1 (0.64%)	2 (1%)	0.363
UVA	2 (4.2%)	1 (0.64%)	3 (1.47%)	0.071
**Duration of follow up**				
Range, months	6-132	6-174	6-174	-
Mean + SD	32.2±35.4	28.3±34.2	29.1±34.6	0.497
**Remission rates**,** n(%)**	20 (42.5%)	67 (42.7%)	87 (42.6%)	0.988
**Time to remission**,** months**,** mean (SD)**	55.4±67.1	47.4±41.3	48.2±49	0.321
**Relapse**,** n(%)**	0 (0%)	7 (4.4%)	7 (3.4%)	0.356
**All-cause mortality rate**,** n (%)**	11 (23.4%)	6 (3.8%)	17 (8.3%)	**< 0.001**

*Other topical treatment included: retinoids, calcipotriol, and crisaborole

**Retinoids included: isotretinoin (*n* = 2), acitretin (*n* = 4)

***Other included: biological medications (secukinumab, *n* = 1), and Janus kinase inhibitors (tofacitinib, *n* = 1). Statistically significant *p*-values are highlighted in bold

Various types of phototherapy were employed in approximately one-third of patients, with PUVA being the most commonly utilized, followed by NBUVB (19.1% and 8.3% of patients, respectively). There was a tendency for a higher rate of phototherapy use in the group associated with malignancy, but the differences were not statistically significant. The follow-up duration after morphea diagnosis ranged from 6 to 174 months, with a mean of 29.1 ± 34.6 months. During the follow-up, 42.6% of patients achieved remission with no significant differences between groups; 3.4% of those relapsed. The all-cause mortality rate was 8.3%, significantly higher in patients with associated malignancy compared to those without (23.4% vs. 3.8%, *p* = 0.00002). Mortality rates differed between patients who developed malignancy before and after morphea diagnosis. Of the 47 patients with both conditions, 11 (23.4%) died during follow-up. Among 29 patients with pre-morphea malignancy, 5 (17.2%) died, while 6 of 23 patients (26%) with pre-malignancy morphea died.

### Analysis of malignancy spectrum, incidence, and timing

Of the 204 patients included in the study, 47 (23%) were diagnosed with malignancy. Within this subset of patients with both morphea and malignancy, 42 (89.4%) had one malignancy, 4 (8.5%) had two malignancies, and one (2.1%) had three malignancies.

The data on malignancy rate, spectrum and timing from the diagnosis of morphea is detailed in Table 3. In 29 (61.7%) of the 47 patients, malignancy occurred prior to the onset of morphea, while in 23 patients (48.9%), it manifested after the diagnosis of morphea. Five patients who presented with multiple types of malignancy experienced occurrences both before and after the diagnosis of morphea.

**Table 3:** The spectrum, incidence, and timing of malignancies in morphea patients

Malignancy type	Patients *n* = 47 f = 37 m = 10	Morphea preceded malignancy (*n* = 23, f = 20, m = 3)		Malignancy preceded morphea (*n* = 29, f = 21, m = 8)	
		Patients, *n* (%)	Time range, years, (mean±SD)	Patients, *n* (%)	Time range, years, (mean±SD)
**Solid organ**					
Breast	12 (25.5%)	2 (8.7%)	3–7(5±2.8)	10 (34.5%)	1–30(7.1±8.4)
Uterus	2 (4.2%)	1 (4.3%)	3	1 (3.4%)	4
Cervix	4 (8.5%)	3 (13%)	1–9(5.3±4)	1 (3.4%)	3
Vulva	2 (4.2%)	1 (4.3%)	7	1 (3.4%)	6
Colon	3 (6.4%)	-	-	3 (10.3%)	1–22(8.5±11.7)
Stomach	2 (4.2%)	2 (8.7%)	1–8(4.5±4.9)	-	-
Renal	1 (2.1%)	-	-	1 (3.4%)	7
Lung	3 (6.4%)	2 (8.7%)	2–10(6±5.6)	1 (3.4%)	6
Thyroid	1 (2.1%)	-	-	1 (3.4%)	48
Prostate	2 (4.2%)	-	-	2 (6.9%)	3–10(6.5±4.9)
Testicle	2 (4.2%)	-	-	2 (6.9%)	2–20(11±12.7)
**Hematological**					
Lymphoma	2 (4.2%)	1 (4.3%)	14	1 (3.4%)	3
Multiple myeloma	2 (4.2%)	1 (4.3%)	6	1 (3.4%)	9
AML	1 (2.1%)	1 (4.3%)	4	-	-
**Cutaneous**					
NMSC	12 (25.5%)	8 (34.8%)	2–8(4.6±2.4)	4 (13.8%)	3–11(6±3.3)
Melanoma	1 (2.1%)	1 (4.3%)	8	-	-

f – female, m – male. AML- acute myeloid leukemia. NMSC – non-melanoma skin cancer

Female predominance was observed in both groups, though not statistically significant (*p* = 0.351). The time interval between the diagnosis of malignancy and morphea exhibited wide variation, spanning from 1 to 30 years. Specifically, in cases associated with breast cancer, when cancer preceded the diagnosis of morphea, the time from cancer onset to morphea ranged from 1 to 30 years, with an average of 7.1 ± 8.4 years. Conversely, when cancer followed the diagnosis of morphea, the time between diagnoses ranged from 3 to 7 years, averaging 5 ± 2.8 years.

### Patients diagnosed with malignancy after morphea diagnosis

In this subgroup, the ages at morphea diagnosis ranged from 10 to 88 with a mean of 67.1 ± 14.6 years. There were no statistically significant differences in age compared to those who developed morphea after a cancer diagnosis (27 to 89 years, mean 63.6 ± 15.0 years; *p* = 0.401). The age range of cancer diagnosis was 31–92 years, with mean of 73.2 (± 12.4) years. The time to cancer diagnosis from morphea onset ranged from 1 to 14 years, with a mean of 5.43 years (± 3.15) years. There were 9 (39.1%) cases of generalized morphea and 14 (60.9%) cases of limited morphea. The most prevalent solid organ malignancy was cervical cancer found in 13% of patients, followed by breast, stomach, and lung cancers, each detected in 8.7% of patients. There was a trend toward a higher rate of non-melanoma skin cancer (34.8% vs. 13.8%) compared to those with malignancy preceding morphea, though it was not statistically significant (*p* = 0.07). Table 4 shows the types of treatment for morphea and the time interval from treatment to cancer diagnosis. Of the patients, 56.5% received only topical treatments, 35% underwent phototherapy, one was treated with methotrexate, and another with etretinate. Of the 8 patients who developed NMSC, 1 received NBUVB, 1 received both NBUVB and BBUVB, 1 received UVA, 1 received PUVA, and 3 did not receive phototherapy. The time period from phototherapy initiation to the development of NMSC ranged from 2 to 6 years. The patient who developed melanoma had previously been treated with PUVA for morphea.

**Table 4:** Treatment modalities for Morphea in 23 patients who developed malignancy post-treatment: a subgroup analysis

Type of treatment	Patients, *n* (%)	Time interval, range (years)	Type of malignancy (*n*)
**Phototherapy**			
NBUVB*	3 (13%)	1–6	NMSC (2) Stomach (1)
UVA**	1 (4.3%)	7	NMSC (1)
PUVA***	3 (13%)	4–8	Breast (1) NMSC (1) Uterus (1)
BBUVB****	1 (4.3%)	2	NMSC (1)
**Systemic pharmacological therapy**			
Methotrexate	1 (4.3%)	1	Cervix (1)
Acitretin	1 (4.3%)	10	Lung (1)
**Topical treatment only**	13 (56.5%)	2–14	NMSC (4) Cervix (2) Hematological (2) Lung (1) Vulvar (1) Breast (1) Melanoma (1) Stomach (1)

*NBUVB-Narrowband ultraviolet B therapy, **UVA-Ultraviolet light A, ***PUVA-Psoralen plus UVA, ****BBUVB-Broadband ultraviolet B therapy. NMSC - non-melanoma skin cancer

### Patients diagnosed with morphea after malignancy

The mean age at cancer diagnosis in this subgroup was 58.6 (± 13.7) years, with 48.2% diagnosed with generalized morphea and 51.8% with limited morphea. Breast cancer was most common in this group, occurring in 34.3% of cases, followed by colon cancer at 10.3%, and prostate and testicular cancers at 6.9% each. Breast cancer was significantly more frequent in this subgroup compared to those diagnosed with morphea before cancer (*p* = 0.028). Among the treatment modalities for all breast cancer patients, all 10 received radiation therapy, one underwent chemotherapy, and four were treated with hormone therapy. Of these 10 patients, 6 had limited plaque-type morphea, and 4 had generalized morphea. In cases with limited plaque-type morphea, it affected sites that were exposed to radiation. One patient with colon cancer received radiotherapy, chemotherapy, and immunotherapy, while two were treated solely with surgery. Table 5 provides details on cancer treatment modalities and the interval between cancer treatment initiation and morphea diagnosis.

**Table 5:** Cancer treatment modalities in 29 patients diagnosed with morphea following malignancy

Cancer treatment modality	Patients, *n* (%)	Time interval, years, range (mean±SD)
Surgery	23 (79.3%)	1–48 (9.46±10.96)
Chemotherapy	4 (13.8%)	2–9 (4.63±3.2)
Radiotherapy	13 (44.8%)	1–30 (6.12±7.58)
Hormone therapy	5 (17.2%)	1–6 (3.6±1.95)
Immunotherapy	2 (6.9%)	2.5-9 (5.75±4.6)
Multimodal treatment*	12 (41.3%)	1–30 (6.96±7.75)

*Multimodal treatment was defined as the use of at least two treatment modalities

## Discussion

Our retrospective cohort study unveils intriguing insights into the potential connections between morphea and various malignancies. While morphea is typically viewed as a dermatological condition, our analysis reveals associations with different types of cancer, as well as the timing of these malignancies relative to the diagnosis of morphea. This analysis provides valuable insights into malignancy types observed in our cohort, their timing in relation to morphea, and potential mechanisms linking these conditions.

Gender distribution in our cohort exhibited a female predominance, consistent with prior studies [1, 2]. However, no significant gender differences emerged between patients with and without malignancy, suggesting gender may not affect cancer risk in morphea patients [1, 12]. Older age emerged as a critical risk factor for cancer in morphea patients, likely due to factors like cumulative genetic mutations, chronic inflammation, and immunological changes [13, 14]. These findings emphasize the importance of monitoring and targeted risk management for older morphea patients.

Significant delays in morphea diagnosis, averaging 3.25 years, were noted, consistent with previous research. These delays, which are likely due to factors such as limited awareness among patients and healthcare providers, restricted access to specialists, nonspecific symptoms, and gradual disease progression, and variable presentations, underline the need for enhanced awareness and improved diagnostics [1, 15]. Importantly, the time to diagnosis didn’t correlate with malignancy status, indicating no significant impact on malignancy development in morphea patients.

In our cohort, the most common malignancies in patients with pre-existing morphea were NMSC, cervical, breast, stomach, and lung cancers, differing from previous findings published by Joly-Chevrier et al. that reported pancreatic, skin, gynecological, and breast cancer as predominant [9]. These discrepancies may stem from geographic variations such as regional dietary habits or smoking rates, as well as methodological differences. Future studies should aim to standardize these factors to enable more consistent comparisons.

NMSC predominated post-morphea diagnosis, consistent with prior research [10, 11]. However, it is important to note that meaningful comparisons of NMSC rates should ideally be made within the same population and age group as the morphea cohort. In our study, extensive UV light exposure emerged as a significant risk factor. Notably, 5 out of the 8 NMSC patients in our cohort had received phototherapy. Although our data suggested a higher tendency for phototherapy use among patients who developed malignancy compared to those who did not, the differences were not statistically significant. Further research is necessary to confirm or refute this observation. Other potential factors contributing to NMSC risk include immunosuppressive therapy, whether used alone or in combination with UV light. Additionally, the persistent inflammation and fibrosis characteristic of morphea, accompanied by increased levels of cytokines such as IL-4, IL-13, and transforming growth factor β, may promote epithelial cell malignant transformation [10, 11]. Genetic predisposition to autoimmune disorders could heighten cancer susceptibility, suggesting a common cause for both conditions [11].

Cervical cancer emerged as the second most frequent malignancy in morphea patients. Supporting this observation, Hemminki K et al. reported an elevated risk of cervical cancer in individuals with localized scleroderma. Factors underlying this association include chronic inflammation and fibrosis, which are known to drive oncogenesis; potential shared genetic or environmental risk factors; hormonal imbalances commonly seen in autoimmune disorders; and immune dysregulation, compromising defense against oncogenic viruses like HPV, thereby heightening the risk of cervical cancer [16]. Additional types of cancer that were more common in morphea patients who developed malignancy after diagnosis included breast, stomach, and lung cancers. Potential explanations include chronic inflammation, fibrosis, and immunosuppressive treatment. It is important to note, however, that Hemminki’s study found no increased risk of breast cancer in morphea patients [16]. The number of cases per type of cancer was small; therefore, there is a need for larger sample sizes. Most patients with cancer did not receive systemic immunosuppressive therapy before their cancer diagnosis, thus indicating that the risk associated with this treatment seems to be less significant. Therefore, further research is needed to clarify this discrepancy.

In our cohort, patients with malignancies preceding morphea, most commonly had breast cancer, NMSC, colon, prostate, and testicular cancers. Breast cancer was the most prevalent, comprising 34.5% of cases. All those with prior breast cancer who developed morphea had received radiation therapy. Among these, 4 cases presented with generalized morphea and 6 with limited plaque-type morphea in irradiated areas, supporting prior studies linking morphea to radiation [17–20]. The relationship between breast cancer and morphea is multifaceted, involving several treatment modalities and biological responses. Radiation therapy, a common treatment for breast cancer, can induce fibrosis by damaging both cancerous and healthy cells, triggering a reactive increase in connective tissue. The tumor itself may also provoke an inflammatory response, contributing to fibrotic changes in the surrounding tissues. Surgical interventions can lead to scarring and fibrosis at the surgical site. Additionally, chemotherapy and hormone therapies used in breast cancer treatment can disrupt normal cellular and hormonal functions, potentially leading to further fibrotic developments. There are also rare cases of morphea linked to chemotherapy and immunotherapy treatments, including PD-1 and CTLA-4 inhibitors [9]. Each of these factors can individually or collectively contribute to morphea onset in breast cancer patients.

Compared to Joly-Chevrier’s review, our cohort’s morphea patients with associated malignancy were notably older (64.7 vs. 38.8 years) and had a shorter time from morphea diagnosis to cancer onset (5.43 vs. 15 years) [9]. In our cohort, although not significant, there was a trend toward higher generalized morphea in the cancer-associated group, consistent with previous findings [9, 21]. Atrophoderma of Pasini and Pierini (APP) was more prevalent in malignancy-associated patients, a novel observation requiring further validation. Research on the association between morphea subtypes and cancer is limited [9–11]. Case reports have linked APP with neoplastic conditions, necessitating additional investigation to elucidate these relationships, particularly regarding Borrelia burgdorferi infections [22–27]. The time interval between cancer treatment and morphea development varied from prior data: 4.6 vs. 1.6 years for chemotherapy, 6.1 vs. 3.1 years for radiotherapy, and 5.7 vs. 0.8 years for immunotherapy.

Increased all-cause mortality in morphea patients with malignancy, particularly those diagnosed before their cancer, necessitates significant clinical focus. Morphea may activate biological pathways predisposing to aggressive or treatment-resistant cancers, a risk heightened by immunosuppressive treatments weakening immune defense. Chronic inflammation linked to morphea could enhance tumor growth, and overlapping symptoms might delay cancer diagnosis. Furthermore, treatments like radiation or chemotherapy could raise secondary cancer risks, with possible inherent genetic or molecular factors in morphea patients amplifying their vulnerability to malignancies.

Strengths of our study include comprehensive data collection from a sizable patient cohort, encompassing diverse demographics to enhance relevance. Detailed clinical evaluations further enrich our analysis. However, limitations include the retrospective nature of the analysis, potentially introducing biases. Conducting the study in a single tertiary care center may limit generalizability, and limited sample sizes for subgroup analyses may reduce statistical power. Reliance on medical records hampers capturing comprehensive patient information, affecting outcomes. While our data indicated certain malignancies were more frequent in specific subgroups, it is important to note that our study was a descriptive analysis within a single cohort, not a case-control study. Therefore, these findings are exploratory and may not be generalizable. The limitations of our retrospective design, including potential biases and the absence of a control group, underscore the need for future case-control studies to confirm or refute our observations and provide stronger evidence on the relationship between morphea and malignancy.

In conclusion, our study suggests potential associations between morphea and malignancies, which appear to be influenced by patient age, sequence of diagnoses, and treatments. Further controlled research is needed to confirm these relationships. If verified, vigilant monitoring, early detection, and refined management strategies will be crucial for morphea patients, especially those with associated cancers.

## Data Availability

No datasets were generated or analysed during the current study.
